# Prevalence and determinants of initiation of breastfeeding within one hour of birth: An analysis of the Bangladesh Demographic and Health Survey, 2014

**DOI:** 10.1371/journal.pone.0220224

**Published:** 2019-07-25

**Authors:** Farhana Karim, Abdullah Nurus Salam Khan, Fariha Tasnim, Mohiuddin Ahsanul Kabir Chowdhury, Sk Masum Billah, Taseen Karim, Shams El Arifeen, Sarah P. Garnett

**Affiliations:** 1 Maternal and Child Health Division, International Centre for Diarrhoeal Disease Research, Bangladesh (icddr,b), Dhaka, Bangladesh; 2 Arnold School of Public Health, University of South Carolina, Columbia, South Carolina, United States of America; 3 Discipline of Child and Adolescent Health, University of Sydney, Camperdown NSW, Australia; Liverpool School of Tropical Medicine, UNITED KINGDOM

## Abstract

**Background:**

Breastfeeding within one hour of birth is a critical component of newborn care and is estimated to avert 22% of neonatal mortality globally. Understanding the determinants of early initiation of breastfeeding (EIBF) is essential for designing targeted and effective breastfeeding promotion programmes. The aim of this study was to determine the prevalence and determinants of early initiation of breastfeeding among Bangladeshi women.

**Methods:**

This paper analyses the data from the Bangladesh Demographic and Health Survey, 2014. Analysis was based on responses of women who had at least one live birth in the two years preceding the survey (n = 3,162) collected using a structured questionnaire. The primary outcome was breastfeeding initiation within one hour of birth ascertained by women’s self-report. Explanatory variables included woman’s age, education, religion, household wealth, place of residence and place of delivery, birth order, child’s size, antenatal care (ANC), postnatal care (PNC) and skin-to-skin contact. Associations between variables were assessed by simple and multivariable logistic regressions.

**Results:**

Of the 3,162 recently delivered mothers, 51% initiated breastfeeding within one hour of delivery. Prevalence of EIBF varied significantly between different types of mode of delivery, among different geographical regions and among women who had PNC with their newborn. Women who had caesarean section (C-section) were less likely to initiate breastfeeding early after birth than women who had normal vaginal delivery (NVD) (AOR: 0.32, 95% CI 0.23 0.43; p value < 0.001). Women who had received PNC with their newborns within one hour of delivery were more likely to breastfeed their babies within one hour of birth compared to those who did not (AOR: 1.61, 95% CI 1.26 2.07; p value < 0.001). Mother’s age, education, religion, household wealth index, place of residence and place of delivery, birth order, number of antenatal visits, child’s size and skin-to-skin contact were not significantly associated with EIBF.

**Conclusions:**

Findings from this study suggest that investing more effort in ensuring immediate PNC of mother-newborn pair can increase EIBF. Solutions should be explored to increase EIBF among mothers who undergo C-section as C-section is rising rapidly in Bangladesh. Further research is needed to explore the regional differences in the country, including specific cultural practices that influence EIBF.

## Background

Early initiation of breastfeeding (EIBF) is recognised as one of the convenient and cost effective intervention strategies to reduce neonatal mortality [1]. The World Health Organization (WHO) and United Nations International Children's Emergency Fund (UNICEF) recommend EIBF, preferably within one hour after birth, and exclusive breastfeeding for the first six months of life [2]. Globally, approximately 22% of neonatal deaths could be averted by ensuring this simple practice [3, 4]. Much effort has gone into promoting breastfeeding including early initiation [5], yet it is estimated that 77 million newborns wait too long to be breastfed after birth [6]. In 1991 the Government of Bangladesh adopted the ‘Baby Friendly Hospital Initiative’, initiated by the WHO and UNICEF, which aims to create an enabling environment at health care facilities for mother and baby [7]. In addition, the Bangladesh Ministry of Health and Family Welfare has incorporated programmes on appropriate breastfeeding practices at the community level [8]. As a result evidence-based breastfeeding practices have increased considerably over the last few decades [9–12]. Although the practice of EIBF within an hour of birth is still low at only 43% and 47% in 2007 and 2011, respectively [10, 11].

Women play the main role in establishing appropriate feeding practices for infants and children since they are generally the primary caregivers [13]. It is important for women to have adequate knowledge and awareness of evidence-based feeding practices for infants and children [14]. In several other studies, number of factors specific to mothers had been identified to have a significant association with the timing of initiation of breastfeeding such as age, education [15–18], religion, place of residence [19, 20] and economic status of their household [21, 22]. Several studies also report on the critical role of antenatal visits and contacts with healthcare provider during immediate postnatal periods on promoting EIBF by counselling the mothers on breastfeeding practices [15–17]. One study examined the association of birth order and child’s size at birth with EIBF and found children who were born large were breastfed early [15]. The literature on EIBF is mostly based on studies conducted at limited setting among specific part of a population which creates an evidence gap at the population level. In this paper we determined the prevalence of breastfeeding initiation within one hour of childbirth i.e. EIBF and examined it’s the association with socio-demographic and geographic factors, and factors related to antenatal care (ANC) seeking, childbirth and immediate postnatal care (PNC) based on data collected at population level in Bangladesh. The results will identify the population characteristics and practices before, during and after childbirth that either favour or avert EIBF at home or in a health facility. The findings will be particularly useful for formulating and implementing effective breastfeeding programmes at scale in the context of low and middle-income countries like Bangladesh.

## Methods

### Study design

This study is based on secondary data analysis of the Bangladesh Demographic and Health Survey (BDHS) 2014, a cross-sectional, nationally representative survey conducted by the National Institute of Population Research and Training (NIPORT) of Bangladesh in collaboration with ICF International (USA), and Mitra and Associates [12]. The survey used a two-stage stratified sampling based on enumeration areas (EA) and household samples. Each EA was considered as the primary sampling unit for this survey and comprise 120 households on average. In the first stage, 600 EAs (urban-207 and rural-393) were selected with probability proportional to the EA size determined from the Population and Housing Census of the People’s Republic of Bangladesh conducted in 2011 [23]. In the second stage, households were selected by systematic sampling from each EA. Details of sampling design, sampling frame and questionnaires used have been published in the final report of BDHS 2014 [12]. Data used in this analysis was collected from married women who had at least one live birth in two years preceding the survey. If multiple babies were born to a mother during this time, information on breastfeeding practices of the last-born infant was collected. The total weighted number of mothers with a live child under two years of age was 3,162. Socio-demographic information and health care seeking practices of mothers were collected by face-to-face interview using a structured questionnaire which was administered during June-November 2014.

### Definition of variables

The outcome variable for this study was ‘early initiation of breastfeeding, EIBF’, defined as initiation of breastfeeding within one hour of birth, according to the recommendations of the WHO and UNICEF [2, 24]. In BDHS 2014, mothers were asked when they started to breastfeed their newborn after birth and answers were documented in numbers of hours, or in days if initiated more than 24 hours after birth [12]. The responses were then categorized into either ‘early’ (within one hour) or ‘late’ initiation (after one hour).

The explanatory variables used in this study were primarily selected based on relevant studies conducted in several low- and middle-income countries including Bangladesh [15–22, 25] and then finalized based on the data available in BDHS 2014. The variables included could be classified into three levels: individual, household and community. The individual-level variables included mother’s socio-demographic characteristics such as age at last birth, education, and religion. Age was categorized into three categories–less than 20 years, 20 to 29 years, and 30 years or more. Mother’s education was categorized into–no education, primary (grade 5) or below and secondary (grade 6) or higher. Information on ANC, birth order of the child, place of delivery, mode of delivery, size of the newborn at birth, skin-to-skin contact of the newborn with the mother immediately after delivery, PNC with a healthcare provider within one hour after delivery were also obtained. The number of ANC visits was recorded as a continuous variable and categorized into ‘no ANC visit’, ‘1–3 ANC visits’ and ‘4 or more ANC visits’. The birth order of the last-born child was categorized into—‘first’, ‘second or third’ and ‘fourth or more’. The place of delivery was categorized into–delivery ‘at health facility’ or ‘at home’ and the mode of delivery was categorized into normal vaginal delivery (NVD) or caesarean delivery. Mother’s perceived size of the newborn was categorized into ‘small’, ‘average’ and ‘large’. PNC was recoded into four categories-no PNC within one hour of delivery, PNC within one hour of delivery received by mother only, newborn only or both mother and newborn. The wealth index was created in two steps. First, the wealth index of mothers’ household, a composite measure of a household's cumulative living standard, was calculated by principal component analysis (PCA) of household's ownership of selected assets during the survey which is a valid technique for creating scores for indices by using both continuous and categorical variables such as ownership of assets [26]. The assets included electricity, televisions, bicycles, ownership of home and livestock; materials that the house was constructed with; access to drinking water and sanitation facilities. The PCA score was categorized into five quintiles- the lowest 20% being the ‘poorest’, the highest 20% being the ‘richest’, and rest of the categories were ‘poorer’, ‘middle’, and ‘richer’. Geographical region and urban or rural residence of mothers’ household were considered as the community level attributes in this survey.

### Statistical analysis

The proportion of women who initiated EIBF is reported as percentages. Simple logistic regressions (unadjusted) were performed between each explanatory variables and outcome variable among the mothers. Variables that showed significant association with EIBF in the simple logistic regressions were included in multivariable logistic regression (adjusted). Mother’s age, education and religion were considered as potential confounders for adjusting in the multivariable regression model. Adjusted odds ratios (AOR) of significantly associated explanatory variables with 95% confidence intervals (CI) are reported. A p-value of <0.05 was considered statistically significant for all analyses performed. Statistical analysis was performed using Stata version 13.0. Sample weight was assigned using ‘*svy*’ command to adjust for clustering effect and sample stratification since BDHS 2014 data was collected non-proportionally from different administrative divisions and urban-rural regions. We used weighted probability that was available in the BDHS dataset.

### Ethical consideration

The 2014 BDHS was conducted under the authority of the National Institute of Population Research and Training (NIPORT) of the Ministry of Health and Family Welfare, Bangladesh. The survey was implemented by Mitra and Associates, a Bangladeshi research organization located in Dhaka. Participants provided consent by signing or a thumb-print if illiterate for the study. The data was made publicly available without any personal identifiers.

## Results

A total of 3,162 mothers reported giving birth within two years preceding the survey and about half (51%) of them initiated breastfeeding within one hour of birth (Table 1). Percentage of EIBF is slightly lower among women of age 30 years or more (49%) than the mothers younger than 30 years. About three-fifth of the women had secondary or higher level of education, however, EIBF was lower among these women (48%) than those who did not have any formal education (56%) or had at least primary level of education (54%). More Muslim mothers initiated the breastfeeding earlier than the non-Muslim mothers (51% vs. 46%, respectively). There is a profound gap in the practice EIBF between the women in the poorest and the richest wealth quintiles (57% vs. 44%, respectively). Majority of women in the study was from the rural region (74%) and more than half of them (53%) initiated breastfeeding within one hour of birth which was higher than those living in urban area (45%). Differences in the proportion of women who initiated early breastfeeding were also observed across different administrative divisions of the country- 60% women from *Rangpur* initiated breastfeeding within one hour of birth, whereas only 39% women from *Khulna* initiated breastfeeding early. EIBF was higher for the child whose birth order was more than 1 than the first-born child. Nearly half of the women (48%) had 1–3 ANC visits and 21% did not have any ANC visit, though 55% of them initiated breastfeeding within one hour of birth. Breastfeeding initiation within one hour of birth was much lower among the women who had C-section (29%) than those had normal delivery (60%) and among those who delivered a health facility compared to those who gave birth at home (39% vs. 59%, respectively). EIBF was also the highest among women who perceived their child as average sized at birth (53%), those who received PNC along with their newborn from a healthcare provider within 1 hour of birth (57%) and those who had skin-to-skin contact with their newborn immediately after birth (54%) compared to their counterparts.

**Table 1:** Percentage distribution of study sample, frequency and prevalence of initiation of breastfeeding within one hour of childbirth by socio-demographic characteristics, and antenatal and perinatal care received by mother.

Characteristics	Total, n (%)	Frequency of breastfeeding initiation within 1 hour of childbirth, n	Prevalence of breastfeeding initiation within 1 hour of childbirth, % (95% CI)
Total	3,162	1613	51.0(49.3–52.8)
**Mother's age at childbirth**			
Less than 20	1002(31.7)	510	50.9(47.8–54.0)
20–29	1742(55.1)	898	51.6(49.2–53.9)
30 or more	418(13.2)	205	49.1(44.2–53.9)
**Mother's education**			
No education	429(13.5)	239	55.6(50.9–60.5)
Primary	894(28.3)	485	54.3(50.9–57.6)
Secondary or higher	1839(58.2)	889	48.4(46.0–50.7)
**Mother's religion**			
Muslim	2923(92.5)	1505	51.5(49.7–53.3)
Non-muslim	239(7.5)	108	45.6(38.8–51.7)
**Wealth index**			
Poorest	686(21.7)	392	57.3(53.3–60.9)
Poorer	609(19.3)	307	50.5(46.4–54.5)
Middle	632(20.0)	321	50.7(46.8–54.8)
Richer	622(19.6)	323	51.9(47.9–55.9)
Richest	613(19.4)	270	43.9(40.1–48.1)
**Place of residence**			
Urban	832(26.3)	378	45.4(42.0–48.9)
Rural	2330(73.7)	1235	53.0(51.0–55.0)
**Division**			
Rangpur	290(9.2)	175	60.3(54.5–66.0)
Barisal	183(5.8)	96	52.4(45.0–59.9)
Chittagong	676(21.4)	309	45.7(41.9–49.6)
Dhaka	1165(36.8)	610	52.4(49.4–55.3)
Khulna	241(7.6)	94	39.0(32.8–45.5)
Rajshahi	319(10.1)	167	52.3(46.7–57.9)
Sylhet	288(9.1)	162	56.3(50.3–62.1)
**Birth Order of child**			
First	1299(41.1)	624	48.0(45.3–50.8)
Second or third	1461(46.2)	777	53.2(50.6–55.8)
Fourth or more	402(12.7)	212	52.7(47.7–57.7)
**Number of ANC visits**			
No ANC visit	648(20.5)	352	54.6(50.4–58.2)
1–3 ANC visits	1518(48.0)	812	53.5(50.9–56.0)
4 or more ANC visits	996(31.5)	449	45.0(42.0–48.2)
**Mode of delivery**			
Normal vaginal delivery	2384(75.4)	1387	58.2(56.2–60.2)
C-section	778(24.6)	226	29.1(25.9–32.4)
**Place of delivery**			
Home	1907(60.2)	1129	59.2(57.0–61.4)
Facility	1255(39.8)	484	38.6(35.9–41.3)
**Child size at birth**^**1**^			
Small	638(20.2)	308	48.2(44.3–52.2)
Average	2148(67.9)	1129	52.6(50.4–54.7)
Large	376(11.9)	176	47.0(41.7–52.0)
**PNC within 1 hour of childbirth**			
Received by none	2210(69.9)	1079	48.8(46.7–50.9)
Received by mother only	113(3.6)	62	55.4(45.2–64.2)
Received by newborn only	142(4.5)	75	52.6(44.3–61.2)
Received by both mother and newborn	697(22.0)	397	57.0(53.2–60.7)
**Skin-to- skin contact**			
No	2303(72.8)	1150	50.0(47.9–52.0)
Yes	859(27.2)	463	53.9(50.5–57.3)

^1^ As perceived by mother

Table 2 presents the crude (unadjusted) and adjusted odds ratios of determinants of EIBF. Household wealth, place of residence, administrative divisions, birth order of the child, number of ANC visits, mode and place of delivery, and PNC within 1 hour were significantly associated with EIBF in simple logistic regression. In multivariable logistic regression the administrative division, mode of delivery and timing of PNC were found significantly associated with EIBF after adjusting for mothers’ age, education and religion. Mothers living in *Chittagong* division (AOR: 0.49, 95% CI 0.35 0.70; p value < 0.001) and *Khulna* division (AOR: 0.50, 95% CI 0.35 0.72; p value < 0.001) were less likely to initiate early breastfeeding compared to mothers living in *Rangpur* division. Mothers who had C-section were less likely to practice EIBF than those who had NVD (AOR: 0.32, 95% CI 0.23 0.43; p value < 0.001). There was 61% increase in the odds of EIBF among the mothers who along with their newborn received PNC within 1 hour of childbirth (AOR: 1.61, 95% CI 1.26 2.07; p value < 0.001) than those who did not receive the care.

**Table 2:** Crude (unadjusted) and adjusted odds ratios of determinants of early initiation of breastfeeding.

Covariates	Breastfeeding initiation within 1 hour of childbirth			
	Crude OR (95% CI)	p-value	Adjusted OR (95% CI)	p-value
**Mother's age at childbirth**				
Less than 20	1.00		1.00	
20–29	1.02(0.82–1.26)	0.848	1.02(0.80–1.31)	0.850
30 or more	0.92(0.69–1.22)	0.574	0.93(0.63–1.38)	0.733
**Mother's education**				
No education	1.00		1.00	
Primary	0.94(0.68–1.30)	0.709	0.96(0.70–1.32)	0.792
Secondary or higher	0.75(0.56–1.00)	0.050	0.97(0.71–1.32)	0.862
**Mother's religion**				
Muslim	1.00		1.00	
Non-muslim	0.81(0.57–1.14)	0.230	0.89(0.62–1.26)	0.512
**Wealth index**				
Poorest	1.00		1.00	
Poorer	**0.74(0.55–0.98)**	0.040*	0.78(0.58–1.05)	0.102
Middle	0.75(0.55–1.02)	0.070	0.97(0.69–1.36)	0.844
Richer	0.80(0.58–1.09)	0.162	1.14(0.80–1.62)	0.454
Richest	**0.57(0.42–0.77)**	0.001*	1.19(0.81–1.75)	0.374
**Place of residence**				
Urban	1.00		1.00	
Rural	**1.35(1.06–1.71)**	0.013*	1.21(0.92–1.60)	0.173
**Division**				
Rangpur	1.00		1.00	
Barisal	0.72(0.50–1.02)	0.065	0.68(0.46–1.01)	0.058
Chittagong	**0.59(0.43–0.80)**	0.001*	**0.49(0.35–0.70)**	0.001*
Dhaka	0.74(0.54–1.03)	0.077	0.76(0.53–1.09)	0.136
Khulna	**0.43(0.31–0.58)**	0.001*	**0.50(0.35–0.72)**	0.001*
Rajshahi	0.73(0.54–0.99)	0.048	0.73(0.52–1.02)	0.063
Sylhet	0.88(0.63–1.25)	0.488	0.75(0.51–1.11)	0.151
**Birth Order of child**				
First	1.00		1.00	
Second or third	**1.23(1.01–1.49)**	0.039*	1.13(0.89–1.44)	0.307
Fourth or more	1.21(0.91–1.61)	0.192	0.93(0.62–1.40)	0.741
**Number of ANC visits**				
No ANC visit	1.00		1.00	
1–3 ANC visits	0.96(0.74–1.23)	0.730	1.15(0.87–1.52)	0.325
4 or more ANC visits	**0.68(0.52–0.89)**	0.005*	1.02(0.74–1.40)	0.909
**Mode of delivery**				
Normal vaginal delivery	1.00		1.00	
C-section	**0.29(0.23–0.37)**	0.001*	**0.32(0.23–0.43)**	0.001*
**Place of delivery**				
Home	1.00		1.00	
Facility	**0.43(0.36–0.52)**	0.001*	**0.77(0.59–0.99)**	0.049*
**Child size at birth**^**1**^				
Small	1.00			
Average	1.19(0.95–1.48)	0.124	-	-
Large	0.95(0.69–1.33)	0.781	-	-
**PNC within 1 hour of childbirth**				
Received by none	1.00		1.00	
Received by mother only	1.32(0.86–2.01)	0.197	1.50(0.97–2.32)	0.070
Received by newborn only	1.17(0.78–1.76)	0.450	1.44(0.97–2.14)	0.073
Received by both mother and newborn	**1.39(1.10–1.77)**	0.006*	**1.61(1.26–2.07)**	0.001*
**Skin-to-skin contact**				
No	1.00		-	-
Yes	1.17(0.96–1.44)	0.124	-	-

^1^ As perceived by mother

*p value <0.05

## Discussion

About half of the surveyed women initiated breastfeeding within one hour of birth (EIBF) in Bangladesh. Factors significantly associated with EIBF practice were the administrative divisions of the country, mode of delivery, and PNC of mother and newborn within one hour after delivery. In contrast to the previous studies, EIBF was not significantly associated with maternal education, place of residence (urban or rural), religion, household wealth, birth order of the child, number of ANC visit, home/facility delivery, child’s size at birth or skin-to-skin contact between mother and newborn immediately after delivery [15–17, 19–22].

The prevalence of EIBF was low at only 51%, but it has increased in the last decade from 23% in 2004, 43% in 2007, and 47% in 2011, which is indicative of a continuing increase of this practice [9–12]. Several other developing countries in South Asia including Nepal, India, and Pakistan observe similar increase in EIBF over the same time period [27–29]. In Bangladesh the practice is higher than that reported in India (36%) [16] and in Pakistan (24%) in 2013 [30]. Several maternal and neonatal health initiatives including promotion of exclusive and early initiation of breastfeeding [31], training of outreach health workers [32], awareness raising campaigns in communities and among opinion leaders [33], and engagement of stakeholders to adapt breastfeeding friendly initiatives in the national nutritional policy [34] are likely to increase the overall breastfeeding practices in the country.

The study identifies that delivery by C-section significantly delays the EIBF among the mothers and this finding is consistent with previous studies including systematic reviews on EIBF [35, 36]. This delay in initiation of breastfeeding is attributed majorly to the practice of separating the newborn from mothers immediately after caesarean childbirth [37]. Distressful condition of mother and critical condition of newborn after C-section also delay breastfeeding initiation after birth [17, 35, 36]. When the situation allows and the newborn is stabilized, promoting skin-to-skin care immediately after caesarean childbirth is an effective way to encourage EIBF [38]. C-section, possibly, is another reason for lower EIBF among mothers who delivered at health facilities than at home as in Bangladesh over 60% of childbirths at health facilities are conducted by C-section [12]. Addressing the reasons for unnecessary C-section and the implementation of strict policies to reduce such unjudicial practices for mothers and their newborn are likely to promote healthy breastfeeding practices. Providing psychological support to these mothers by family members and healthcare providers can create a supportive environment for EIBF even in critical conditions like C-section as reported in a Cochrane review [39] and promoted by Baby Friendly Hospital Initiative (BFHI) [40].

Our results show that the odds of EIBF is 61% higher among women who along with their child received postnatal care from healthcare provider within one hour of birth. This practice is strongly supported by the WHO recommendation- postnatal visits should happen as early as possible after birth [41]. The early postnatal period is critical for developing the bonding between the mother and her newborn and allows the attending healthcare provider to provide counselling or support to initiate breastfeeding properly [42]. Provision of training to the birth attendants and other obstetric health workers including nurses and midwives on breastfeeding promotion, education and counselling can be critical for increasing the EIBF. Evidence shows that the presence of a breastfeeding- trained delivery assistant during childbirth increases the rates of EIBF in Nigeria [37]. Moreover, educating the family members and peer group to support mother immediately after childbirth should be prioritized to increase appropriate breastfeeding practices including EIBF. A Lancet study shows that educating local mothers to act as a peer for counselling recent mothers in their community could increase the breastfeeding practices among them [33].

In this analysis, timely initiation of breastfeeding was not significantly associated with maternal education or urban/rural residence of the mother. These findings are contrary to the findings of several other studies in developing countries which have indicated that EIBF was more likely to be practised by educated, urban mothers from the richer household [15, 19, 43]. This is possible that mothers in Bangladesh are aware of the good breastfeeding practices irrespective of their socio-economic status through interpersonal communication during home visits, community-based campaigns including group discussions and counselling session at heath centres, or exposure to messages through mass media [44]. Nonetheless, the awareness raising activities should continue to sustain high diffusion of promotional messages among the disadvantaged part of the country.

Breastfeeding counselling in the third trimester of pregnancy and regular ANC visits have been shown to be associated with increased EIBF [45, 46]; however, our study does not find a significant relationship between EIBF and increased frequency of ANC. This could be explained by the content of ANC in Bangladesh and other LMICs where messages on breastfeeding practices are rarely communicated with pregnant women [47]. As the ANC coverage in Bangladesh is gradually increasing, we should engage the ANC providers to counsel the pregnant women on early and exclusive breastfeeding especially at the later trimester of pregnancy. Initiation of breastfeeding within one hour after birth was significantly lower in *Chittagong* (46%) and *Khulna* (39%) divisions in Bangladesh compared to other divisions, which is consistent with previous national health surveys [10, 11]. The low proportion in *Chittagong* could be attributed to the scattered nature of dwellings of many tribal populations in this area. They have poor communication with the main land, frequently move for cultivation, have multiplicity of ethnicities and languages and geographical variations (hilly terrain and forest areas) and all these attributes make the implementation of health programmes very difficult [48]. In Khulna division, childbirth by C-section remains high compared with other divisions of the country which might influence low EIBF practice. Further research is needed to explore the differences in region-specific cultural practices which could be associated with EIBF to improve the effectiveness of breastfeeding program in the country.

An important strength of this study is that it used a large nationally representative sample. Both the study methodology and the questionnaire used in the survey were validated. There are few limitations; first, the recall-based responses with regard to the breastfeeding initiation time after birth might introduce error in reporting by mothers. Since giving birth is an important event for the mothers, we assume such error would occur among the minority of the sample and the overall result will less likely be biased given the large dataset. Some potential factors and barriers for EIBF such as mother’s knowledge on breastfeeding, breastfeeding counselling during ANC, the timing of ANC, social support could not be explored in this analysis due to unavailability of such data in the survey.

## Conclusions

Uptake of early initiation of breastfeeding is a vital strategy to avert a significant number of newborn deaths and to achieve the global target of reducing neonatal mortality rate to at least as low as 12 deaths per 1,000 live births by 2030. This paper depicts the importance of PNC within an hour of delivery for EIBF. Attendance of healthcare providers with adequate knowledge about infant feeding practices during this time could convey and encourage key feeding practices. The relevant stakeholders and policymakers should consider this fact for taking policy-level decisions to ensure early PNC through special training packages and community mobilization. Besides, early breastfeeding initiation after C-section should be focused on designing policies to promote appropriate feeding practices of the newborn. Further research on the factors associated with EIBF is warranted so that specific programmes can be designed to increase the practice and save newborn lives.
